# Editorial: The Pathogenic Yersiniae–Advances in the Understanding of Physiology and Virulence, Second Edition

**DOI:** 10.3389/fcimb.2019.00119

**Published:** 2019-04-18

**Authors:** Matthew S. Francis, Victoria Auerbuch

**Affiliations:** ^1^Department of Molecular Biology, Umeå Centre for Microbial Research, Umeå University, Umeå, Sweden; ^2^Department of Microbiology and Environmental Toxicology, University of California, Santa Cruz, Santa Cruz, CA, United States

**Keywords:** phenotypic and niche adaptation, protein secretion, biofilm, small regulatory RNAs, two-component systems, virulence blockers, Crohn's disease, fish- and insect-pathogen

Of the 18 known *Yersinia* species, *Y. pestis, Y. pseudotuberculosis*, and *Y. enterocolitica* are pathogenic to humans and animals and are widely characterized. The zoonotic obligate pathogen *Y. pestis* is the causal agent of plague, a systemic disease that is usually fatal if left untreated (Zietz and Dunkelberg, 2004; Zhou et al., 2006). Free-living *Y. enterocolitica* and *Y. pseudotuberculosis* are the agents of yersiniosis, a rarely systemic gastrointestinal disease (Galindo et al., 2011). The remaining species are mostly harmless to humans, although *Y. ruckeri* is an enteric fish pathogen affecting mainly salmonids, while a few others display toxicity toward insects (Sulakvelidze, 2000; Tobback et al., 2007; Fuchs et al., 2008; Chen et al., 2010). At the forefront of *Yersinia* research are studies of classical microbiology, pathogenesis, protein secretion, niche adaptation, and regulation of gene expression. In pursuit of these endeavors, new frontiers are being forged on waves of methodological and technological innovation. In this second edition of the special research topic on the pathogenic Yersiniae is a compilation of reviews and research articles that summarize current knowledge and future research directions in the *Yersinia* pathophysiology field.

## Protein Secretion

Type III secretion (T3S) is prominent protein delivery process in a large number of Gram-negative bacteria that confers to them an ability to interact in pathogenic or symbiotic relationships with either vertebrate or invertebrate hosts (Buttner, 2012; Deng et al., 2017). The Ysc-Yop T3S system (T3SS) is encoded on a virulence plasmid common to all human pathogenic *Yersinia* (Cornelis et al., 1998). This so-called “injectisome” has long been believed to provide a conduit through which a restricted set of just six or seven plasmid-encoded host-modulating Yop effectors are delivered from the bacterial cytoplasm into the eukaryotic cell cytosol (Pha and Navarro, 2016; Grabowski et al., 2017). Using a transposon site hybridization based genome wide screen, Schesser-Bartra et al. identified three chromosomally-encoded proteins that promote *Y. pestis* infection in cells and in mice. With features indicative of host-modulating Yop effectors, they identify the first non-plasmid encoded secretion substrates of the Ysc-Yop T3SS. In another study that performed heterologous complementation analyses with the YscX and YscY protein families, Gurung et al. reveal that the YscX and YscY protein complex produced by *Y. pseudotuberculosis* is specifically critical for biogenesis and/or function of the Ysc-Yop T3SS. The authors go on to discuss what might be the molecular basis for this specificity.

While pathogenic potential of *Yersinia* for humans and animals is heavily correlated to the plasmid encoded Ysc-Yop T3SS, Yang et al. provide new insight into the four independent Type VI secretion system (T6SS) copies present in human pathogenic *Yersinia*. The impact of these multiple systems on *Yersinia* physiology and pathogenesis is likely to be very large given how T6SSs have capacity to deliver multiple effectors into either prokaryotic or eukaryotic cells, and are known to affect diverse biological processes such as virulence, anti-virulence, stress resistance and competition (Alteri and Mobley, 2016; Lien and Lai, 2017).

## Niche Adaptation

Enteropathogenic *Yersinia* are foodborne pathogens. Therefore it comes as no surprise that they thrive at refrigeration temperatures (Brocklehurst and Lund, 1990; Goverde et al., 1994; Azizoglu and Kathariou, 2010; Keto-Timonen et al., 2018), and in this environment even remain primed for infection (Asadishad et al., 2013). To understand the molecular mechanisms by which psychotropic *Yersinia* thrive in cold environments may give rise to strategies by which growth can be restricted, and this would be a strategically important preventative measure for the food processing industry. To investigate the genome-wide cold adaptation behavior of *Y. pseudotuberculosis*, Virtanen et al. used RNA-Seq technology to identify genes that were significantly more expressed in a cell density specific manner at cold temperature. Among the many genes that were up-regulated were nutrient acquisition genes, cold shock protein genes, DEAD-box RNA helicase genes, genes handling compatible solutes, genes involved in transcription termination and translation initiation, and genes involved in cell wall modification. This suggests that *Y. pseudotuberculosis* establishes a core network of cold responsive proteins to drive ribosome biogenesis and function at low temperature.

It follows that psychotropic *Yersinia* are enriched in a variety of foods on a global scale (Hilbert et al., 2003; Ozdemir and Arslan, 2015; Le Guern et al., 2016). Moreover, changing food consumption practices and globalization of the international food trade have contributed to increased frequency of yersiniosis (Gupta et al., 2015). At the same time, orally ingested *Yersinia* have the potential to survive passage through the gastrointestinal tract. It has been postulated that surviving bacteria may contribute to the onset or persistence of gut inflammation (Hugot et al., 2003). Although experimental mouse models of Crohn's disease do not discount contributions made by infecting enteropathogenic *Yersinia* (Meinzer et al., 2008; Murthy et al., 2014; Fonseca et al., 2015; Han et al., 2017), support stemming from cohort studies of *Yersinia* infected clinical material is underwhelming (Kallinowski et al., 1998; Lamps et al., 2003; Knosel et al., 2009; Chiodini et al., 2013; Leu et al., 2013). To further investigate this issue, Le Baut et al. analyzed the prevalence of *Yersinia* species in a total of 470 illeal samples taken from Crohn's disease patients and healthy controls. Significantly, *Yersinia* species were detected with equal frequency in both disease and healthy ileum tissue, suggesting that they are well adapted to this niche. Hence, there is now a need to characterize the effect of resident *Yersinia* on maturation and regulation of the mucosal immune response.

## Gene Expression Control

Behind every successful niche adaptation is a complex regulatory circuitry that controls specific gene expression profiles. For example, the two-component or histidine-aspartate phosphorelay systems are vital for the monitoring of environmental and intracellular signals to produce changes in gene expression or behavioral responses (Stock et al., 2000; Laub and Goulian, 2007). In *Yersinia*, a large number of two-component systems are known (Marceau, 2005), with a few of them making recognized contributions to *Yersinia* survivability in the environment or in an infected host (Flamez et al., 2008; Reboul et al., 2014). A notable two component system is EnvZ/OmpR that enables many bacteria to alter gene expression in response to osmotic and acid stress (Walthers et al., 2005; Chakraborty and Kenney, 2018). The work of Jaworska et al. reports on OmpR-mediated control of iron acquisition via transcriptional repression of the HemR1 and HemR2 heme receptors. This regulatory circuit works in conjunction with the transcriptional repressor Fur to prevent over-accumulation of iron/heme by *Y. entercolitica*.

Another two component system is BarA/UvrY. Responding to metabolic end products such as short chain fatty acids, BarA/UvrY signaling is the primary regulator of the widespread Csr global regulatory system, and in this way can profoundly influence multiple metabolic, behavioral and virulence traits in many bacteria (Vakulskas et al., 2015). In the report by Schachterle et al. BarA/UvrY signaling was found to repress the formation of *Y. pseudotuberculosis* biofilms through activation of the CsrB regulatory RNA. It is likely that this is pleiotropic repression affecting multiple elements of biofilm formation and maintenance by *Y. pseudotuberculosis*.

The primary requirement for mature biofilm formation by *Yersinia* is the production of an exopolysaccharide (EPS) that requires the *hmsHFRS* locus to coordinate its synthesis and transport (Bobrov et al., 2008). Moreover, c-di-GMP enhances EPS production, and the levels of this signaling molecule are tightly controlled by the opposing actions of two diguanylate cyclases (encoded by *hmsT* and *hmsD*) and a phosphodiesterase (*hmsP*) (Kirillina et al., 2004; Bobrov et al., 2011). The study of Fang et al. describes a novel AraC-like transcriptional activator termed BfvR that controls *Y. pestis* biofilm formation via stimulating transcription from the *hmsHFRS* and *hmsCDE* operons to elevate EPS and c-di-GMP production. This identifies BfvR as the first AraC family transcription regulator reported to control biofilm formation in *Yersinia*.

## Microbiology and Pathogenesis of Non-Mammalian *Yersinia* Infections

Although not known to be harmful to humans, the enteric fish pathogen *Y. ruckeri* is still a pathogen of great interest as it has capacity to causes significant economic losses in the aquaculture industry (Tobback et al., 2007). This is reflected by a recent surge of reports that offer improved understanding of the biological processes contributing to *Y. ruckeri* infection and pathogenicity. The review by Guijarro et al. assimilates this new knowledge to provide up-to-date insight into the molecular mechanisms of the *Y. ruckeri* infection process. Complementing this review is a report by Wrobel et al. that analyzed the complete DNA sequence of the unique pYR4 plasmid from a highly virulent isolate of *Y. ruckeri*. This cryptic plasmid has potential to impact positively on *Y. ruckeri* virulence since it encodes for a type IV pilus and a type IV secretion system that are well established virulence associated factors in other bacteria (Craig et al., 2004; Giltner et al., 2012; Gonzalez-Rivera et al., 2016; Grohmann et al., 2018).

Moreover, there has been great interest in the function and taxonomical distribution of insecticidal genes among *Yersinia* spp., owing in part to their potential in contributing new knowledge to the ecology, evolution and pathogenicity of human pathogenic *Yersinia* (Pinheiro and Ellar, 2007; Fuchs et al., 2008, 2011; Hares et al., 2008; Spinner et al., 2012, 2013; Alenizi et al., 2016). Using *Y. frederiksenii* as a model system that displays toxicity toward insects, Springer et al. were able to demonstrate a distinct contribution of the novel heat-stable cytotonic enterotoxin to oral and intrahemocoelic toxicity of infected insects. These findings led the authors to discuss how the ability to enter invertebrates may constitute a selective advantage to *Yersinia* isolates in environmental survival and evolution of virulence.

## New Frontiers in *Yersinia* Biology Research

Conventional antibiotics have saved the lives of many by decreasing the morbidity and mortality of bacterial infectious diseases. However, the global emergence of bacteria resistant to these antibiotics means that they no longer work effectively, and this presents a major healthcare issue that creates tremendous global social and economic suffering (Aminov, 2010). Consequently, alternative solutions to this healthcare crisis that are effective and reliable must be swiftly identified. In recent years, one such alternate approach has been to isolate anti-bacterials that function by targeting a virulence determinant (Clatworthy et al., 2007; Maura et al., 2016). Ideally, these so called “anti-infectives” or “virulence blockers” would be specific for pathogenic bacteria and have a bacteriostatic effect that would synergize with the immune system to clear the infection. A classic example of this endeavor is the identification of novel chemical inhibitors of the T3SS (Keyser et al., 2008; Duncan et al., 2012). Despite the success of identifying chemical inhibitors of the T3SS, none of these have yet reached the market. This issue is addressed in a report by Morgan et al. which describes the development of an experimental pipeline that would help transition from high throughput screening to inhibitor validation and initial determination of their mode of action. In so doing, the authors consider important new possible modes of action for T3SS inhibitors.

Bacterial virulence regulation is exquisitely fine-tuned so that subsets of virulence factors are expressed only at times of need. Alterations in the local environment account for triggering changes in this virulence gene expression profile. Responses are rapid, and it is now clear that post-transcriptional regulatory effects, such as small non-coding RNAs contribute to the rapidity of this re-programming. Benefitting from progressive developments in genome-wide omics-based methods of exploration, several RNA-based regulatory systems have been discovered in pathogenic *Yersinia*. These discoveries have been reviewed by Knittel et al. in the context of *Yersinia* niche colonization, metabolic adaptation, acute and chronic infection, and evolution. By inference, at least some RNA-based regulatory systems could serve as a suitable target of anti-infective drug development.

The second edition of this research topic, provides many examples demonstrating the great capacity of *Yersinia* species to adapt and thrive in diverse environmental niches. This is reiterated by the timely review by Davis, which sheds light on the ability of *Yersinia* sub-populations to phenotypically diversify during an infection in order to balance the need to maintain bacterial growth while resisting attack from different cellular elements of an activated immune system. Underpinning this phenotypic diversification is the ability of subsets of bacteria to make temporal and spatial adjustments to their gene expression profiles in response to the microenvironment. Having the technology to detect gene expression profiles in distinct sub-populations of bacteria offers a unique opportunity to understand the yin and yang of interactions between individual bacteria and specific immune cell types. In turn, this may eventually enable the generation of more efficacious approaches to treat infections by having the option of tailoring novel antibacterials or their immunomodulatory counterparts that can favorably influence the outcome of this bacteria-immune cell interplay.

## Author Contributions

MF developed the initial concept and outline. Both MF and VA contributed to the final version of the manuscript.

### Conflict of Interest Statement

The authors declare that the research was conducted in the absence of any commercial or financial relationships that could be construed as a potential conflict of interest.
